# Impact of Automated Blood Culture Systems on the Management of Bloodstream Infections: Results from a Crossover Diagnostic Clinical Trial

**DOI:** 10.1128/spectrum.01436-22

**Published:** 2022-09-12

**Authors:** Ana Verónica Halperin, Juan Antonio del Castillo Polo, José Luis Cortes-Cuevas, María José Cardenas Isasi, Mario Ampuero Morisaki, Robert Birch, Ana María Sánchez Díaz, Rafael Cantón

**Affiliations:** a Servicio de Microbiología, Hospital Universitario Ramón y Cajal, Madrid, Spain; b Instituto Ramón y Cajal de Investigación Sanitaria (IRYCIS), Madrid, Spain; c Data Analytics, bioMérieux, Hazelwood, Missouri, USA; d CIBER de Enfermedades Infecciosas (CIBERINFEC), Instituto de Salud Carlos III, Madrid, Spain; Instituto de Higiene

**Keywords:** blood culture, clinical diagnostic trial, time to detection, turnaround time

## Abstract

Bloodstream infections are associated with high rates of morbidity and mortality. Blood culture remains the gold standard for the diagnosis of BSIs. We report a prospective crossover diagnostic clinical trial comparing the performances of two blood culture incubation systems: Virtuo and Bactec FX. The primary outcome was the time to detection (TTD) (from the loading of the sample into the incubator to the positivity signal). Patients over 16 years old suspected of having bacteremia/fungemia were included. They were divided into two strata with a total of 9,957 blood extractions. Initially, each stratum was randomly assigned to one of the incubators and then alternated every 2 weeks for 6 months. Each sample was inoculated into an aerobic bottle and an anaerobic bottle. All bottles were processed equally according to the laboratory’s standard procedures after they were flagged positive. We analyzed 4,797 samples in the Virtuo system and 5,160 in the Bactec FX system. The median TTD was significantly lower for the Virtuo group (Virtuo, 15.2 h; Bactec FX, 16.3 h [*P* < 0.0001]). The turnaround time (TAT) (from sample loading to the Gram stain report) was also reduced with Virtuo (Virtuo, 26.2 h; Bactec FX, 28.3 h [*P* < 0.004]). When considering only samples from patients with antimicrobial treatment prior to blood culture extraction, the TTD was shorter for Virtuo (median differences in the TTD of 4.5 h for all bottles and 8.7 h for aerobic bottles only [*P* = 0.0001]). In conclusion, virtuo provided shorter TTD and TAT than Bactec FX. The difference in the median TTD was increased when considering samples incubated in aerobic bottles from patients with antimicrobial treatment. This could have an important effect on the faster diagnosis of BSIs.

**IMPORTANCE** Bloodstream infections are associated with high rates of morbidity and mortality. Blood culture remains the gold standard for its diagnosis. While the identification of the pathogen and its antibiotic susceptibility is required to confirm the optimal antimicrobial regimen, reductions in the times to the detection of positivity and reporting of Gram stain results may be important and time-saving to reduce inappropriate antimicrobial use, improve patient outcomes, and decrease health care costs. We report the first clinical diagnostic study of this scale in a “real-world” setting with a crossover design, comparing two automatic blood culture incubators using samples from patients with a suspected diagnosis of bacteremia/sepsis, as opposed to spiked vials. Our study design mimics that of clinical trials performed for drug marketing authorization, but patient randomization was replaced with the crossover design. A shorter time to detection could have an important effect on the faster identification of causative microorganisms of BSIs and antimicrobial stewardship.

## INTRODUCTION

Throughout the world, the number of patients at risk of bloodstream infections (BSIs) continues to rise (1, 2). BSIs are associated with high rates of morbidity and mortality and markedly increase the costs of hospital care. Furthermore, there is a persistent impaired quality of life and higher rates of long-term mortality among hospital survivors after septic shock (3, 4). The prompt identification of the causative agent(s) and the rapid initiation of appropriate antimicrobial therapy are critical for reducing mortality, especially in patients with septic shock (5, 6). Blood culture (BC) remains the gold standard for the diagnosis of BSIs (7).

Over the decades, improvements in BC media combined with the availability of automated growth detection have enhanced the recovery of bloodstream pathogens and decreased the time to detection (TTD) of bacterial growth. Various continuously monitored BC systems, based on colorimetric (BacT/Alert; bioMérieux, Marcy l’Etoile, France) or fluorescence (Bactec; Becton, Dickinson Instrument Systems, Sparks, MD, USA) detection of CO_2_ produced by replicating microorganisms, are used extensively in clinical microbiology laboratories to detect the causative agent(s) of BSIs. Both systems employ resin-containing media in BC bottles (i.e., BacT/Alert FAN Plus or Bactec FX Plus) to enhance organism recovery. Most clinical laboratories commonly pair aerobic and anaerobic BC bottles to better recover the vast array of blood pathogens.

In the context of BSIs, while the identification of the pathogen and its antibiotic susceptibility is required to confirm the optimal antimicrobial regimen, reductions in the TTD and the reporting of Gram stain results may be important and time-saving to reduce inappropriate antimicrobial use, improve patient outcomes, and decrease health care costs. A study examining the impact of clinical microbiology laboratory services on the management of clinically significant BSIs found that 75% of antimicrobial regimen initiations took place following the Gram stain report from a positive BC and that notification to the clinician of such results had a greater impact on antimicrobial usage than the provision of antimicrobial susceptibility testing (AST) results (8). In addition, the implementation of targeted antibiotic therapy has been demonstrated to be directly associated with the time required to obtain results; modifications to antibiotic regimens are less likely to occur when the time to results is longer (9). Consequently, faster reporting of positive blood culture results is critical for optimal patient management and reduced health care costs.

Here, we report the results of a prospective crossover diagnostic clinical trial that compared the performances of two different blood culture incubation systems in a real-life setting (BacT/Alert Virtuo and Bactec FX) with patient samples from a tertiary hospital with a 24/7 microbiology laboratory. The primary outcome was the TTD. The secondary outcomes were the turnaround time (TAT) and hands-on-time performance. To our knowledge, this is the first time that this approach has been used to compare two diagnostic systems.

## RESULTS

We analyzed a total of 9,957 extractions, 4,797 in the Virtuo arm and 5,160 in the Bactec FX arm (9,538 and 10,204 bottles, respectively).

**FIG 1:**
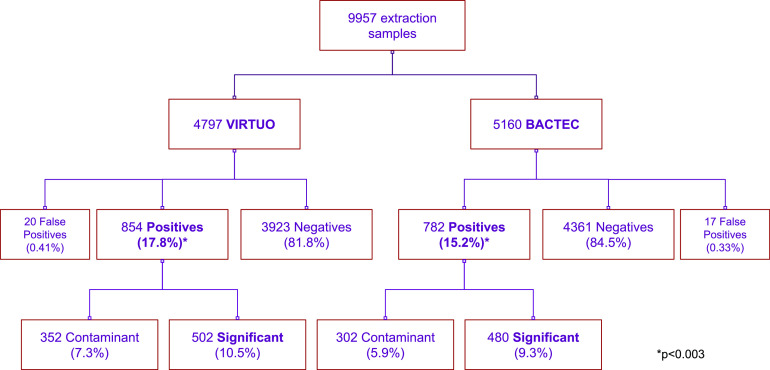
Sample distribution.

The median age of the population was 67 years (interquartile ranges [IQRs], 55 to 83 years for Virtuo and 53 to 83 years for Bactec FX) for both groups, and the origins of the samples were similarly distributed for each incubator (emergency department [ED] 90.7% in the Virtuo group and 87% in the Bactec FX group). In the Virtuo group, 926 (53%) of the subjects were male, while in the Bactec group, 1,049 were male (55.8%) (*P* = 0.0917). The numbers of blood cultures with at least one positive extraction were 874 (18.2%) for Virtuo and 799 (15.5%) for Bactec FX (*P* = 0.0003), including false-positive results (Virtuo, 20; Bactec FX, 17). Differences in the detection of significant microorganisms when excluding contaminants were not statistically significant between the groups (*P* = 0.052). For significant microorganisms only, the proportion of anaerobic bottles to be flagged positive first was no different between groups (Virtuo, 5.5%; Bactec FX, 5.8% [*P* = 0.45]); however, it was larger in the Virtuo group for aerobic bottles (Virtuo, 5.0%; Bactec FX, 3.6% [*P* = 0.0005]). The distribution of the samples is shown in Fig. 1.

**FIG 2:**
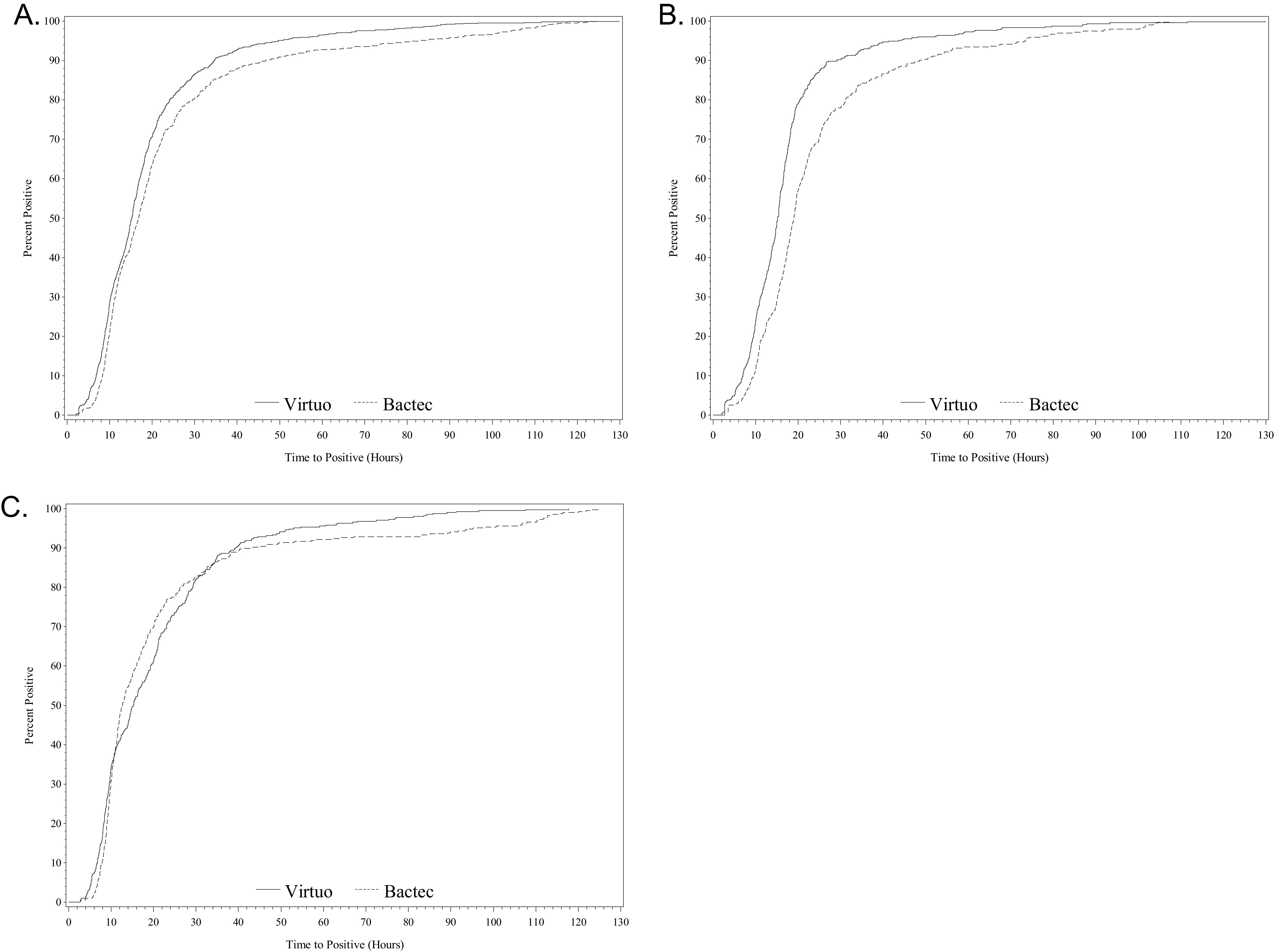
TTD by instrument for all positive bottles. (A) All positive bottles (Virtuo, *n* = 854; Bactec FX, *n* = 782) (*P* < 0.0001). (B) Aerobic bottles (Virtuo, *n* = 459; Bactec FX, *n* = 381) (*P* < 0.0001). (C) Anaerobic bottles (Virtuo, *n* = 406; Bactec FX, *n* = 408) (*P* = 0.7503).

Overall, the median TTD per blood culture extraction, including all bottle types, was significantly shorter in the Virtuo group (Table 1 and Fig. 2). The TTD was also shorter in the Virtuo arm when analyzing significant organisms and contaminant results separately (Table 1). The difference in the TTD was greatest for aerobic bottles incubated in the Virtuo system, with reductions in the median TTD of 3.7 h and 4.1 h for significant organisms and contaminants, respectively. Even though the TTD was shorter for all positive anaerobic bottles incubated in the Bactec FX system, this difference did not reach statistical significance.

**TABLE 1:** Time to detection

Sample type	Median TTD (h) (IQR)		*P* value^*a*^
	Virtuo	Bactec FX	
All positive			
All bottles	15.2 (9.6–21.5)	16.7 (10.6–25.4)	**<0.0001**
Aerobic	15.2 (10.2–18.9)	19.0 (13.6–27.0)	**<0.0001**
Anaerobic	15.3 (8.9–25.9)	12.8 (9.6–22.3)	0.7503
Contaminants			
All bottles	18.9 (15.5–26.5)	22.3 (18.4–33.1)	**<0.0001**
Aerobic	17.2 (15.0–21.0)	21.3 (18.1–27.8)	**<0.0001**
Anaerobic	24.8 (19.3–34.8)	26.2 (20.2–49.2)	**0.0202**
Significant organisms			
All bottles	10.6 (8.2–17.0)	11.6 (9.3–17.5)	**0.0060**
Aerobic	11.2 (8.7–16.7)	14.9 (10.4–25.1)	**<0.0001**
Anaerobic	10.0 (8.0–17.4)	10.7 (8.9–14.6)	0.7168

a Statistically significant values are indicated in boldface type.

Table 2 summarizes the TTD and TAT by patient characteristics and microorganisms. The median TAT was reduced by 2.1 h for samples incubated in the Virtuo system (*P* = 0.004). For aerobic bottles only, the median TAT was shortened by 9 h when samples were incubated in the Virtuo system compared to Bactec FX (*P* < 0.0001), while no statistically significant difference was observed for anaerobic bottles. When analyzing significant organisms only, the median TAT for samples incubated in the Virtuo system was 22.3 h, and that for samples incubated in the Bactec FX system was 23.0 h (*P* > 0.05).

**TABLE 2:** Time to detection and turnaround time by patient characteristics^*a*^

Characteristic	Virtuo		Bactec FX		Subgroup analysis for positive extraction with TAT available^*b*^					
	No. of positive extractions	Median TTD (h)	No. of positive extractions	Median TTD (h)	Virtuo			Bactec FX		
					No. of extractions with TAT available	Median TTD (h)	Median TAT (h)	No. of extractions with TAT available	Median TTD (h)	Median TAT (h)
Overall	852	15.3	782	16.7*	842	15.4	26.0	767	16.9*	28.4*
Ward										
Stratum 1 (ED)	697	14.8	619	16.0*	687	15.0	25.5	609	16.3*	27.8*
Stratum 2 (other wards)	155	17.0	163	19.2*	155	17.0	27.7	158	19.2*	31.5*
Bottle detected first^*c*^										
Aerobic	453	15.4	378	19.2*	445	15.2	25.5	377	19.1*	35.0*
Anaerobic	399	15.3	404	12.6	397	15.4	26.8	390	12.8	24.5
Gram stain result										
Gram negative	261	9.6	258	10.2	257	9.5	21.7	254	10.3*	20.8
Gram positive	553	17.4	483	20.0*	549	17.7	32.4	477	19.9*	36.6*
Organism type										
Contaminant	352	18.9	302	22.3*	347	18.9	38.9	296	22.4*	41.0*
Significant organism	500	10.6	480	11.6*	495	10.7	22.4	471	11.6*	23.0
Hospital-acquired infection										
No	737	14.9	680	16.4*	727	15.0	25.6	670	16.6*	28.4*
Yes	114	18.4	95	18.6*	114	18.4	30.3	90	18.4*	27.7
Infection acquired <90 days from previous discharge										
No	655	15.4	574	16.7*	649	15.5	26.7	565	16.9*	30.4*
Yes	196	14.6	201	16.3*	192	14.7	24.7	195	16.3*	27.0*
Specific organism(s)										
CoNS	62	15.4	39	17.1	61	15.4	30.2	38	17.6	26.6
Escherichia coli	162	8.5	169	9.6*	158	8.5	18.1	165	9.6*	19.0
Klebsiella pneumoniae	48	9.6	29	9.2	44	9.9	20.1	25	9.2	21.3
Staphylococcus aureus	55	14.7	48	15.6	53	14.8	22.0	48	15.6	34.7*
qSOFA score (significant organisms only)										
0	286	10.8	256	11.5	282	10.9	22.5	250	11.5	22.9
1	137	10.2	143	11.1	132	10.6	21.7	141	11.2	23.5
≥2	58	9.9	65	11.4*	58	9.9	22.3	61	12.3*	20.8

a *, *P* < 0.05 for Virtuo versus Bactec FX.

b For samples with no record of the time at which the Gram stain result was reported, the TAT was not calculated.

c One culture for Virtuo was missing bottle type data for two extractions. Seven cultures had aerobic and anaerobic bottles positive at the same time. They are included in both the aerobic and anaerobic portions of the table. ED, emergency department; CoNS, coagulase-negative staphylococci; h, hours.

For patients identified as being more likely to have poor outcomes typical of sepsis by a quick sequential organ failure assessment (qSOFA) score of ≥2 (respiratory rate of 22 breaths per minute or higher, altered mental status, or systolic blood pressure of 100 mm Hg or lower) (10), a reduction in the TTD was statistically significant in favor of Virtuo.

The median time to pathogen identification (time to ID) and time to AST results were not statistically significantly different between groups when all bottle types were pooled (46.9 h for Virtuo and 46.5 h for Bactec FX [*P* = 0.1921] for time to ID; 58.1 h for Virtuo and 58.5 h for Bactec FX [*P* = 0.33] for time to AST results). However, when comparing only aerobic bottles, the median time to ID and the median time to AST results were reduced by 6.7 h and 6.6 h, respectively, when samples were incubated in the Virtuo system (median times to ID of 45.5 h for Virtuo and 52.2 h for Bactec FX [*P* < 0.0001]; median times to AST results of 54.4 h for Virtuo and 61.0 h for Bactec FX [*P* < 0.0001]).

**FIG 3:**
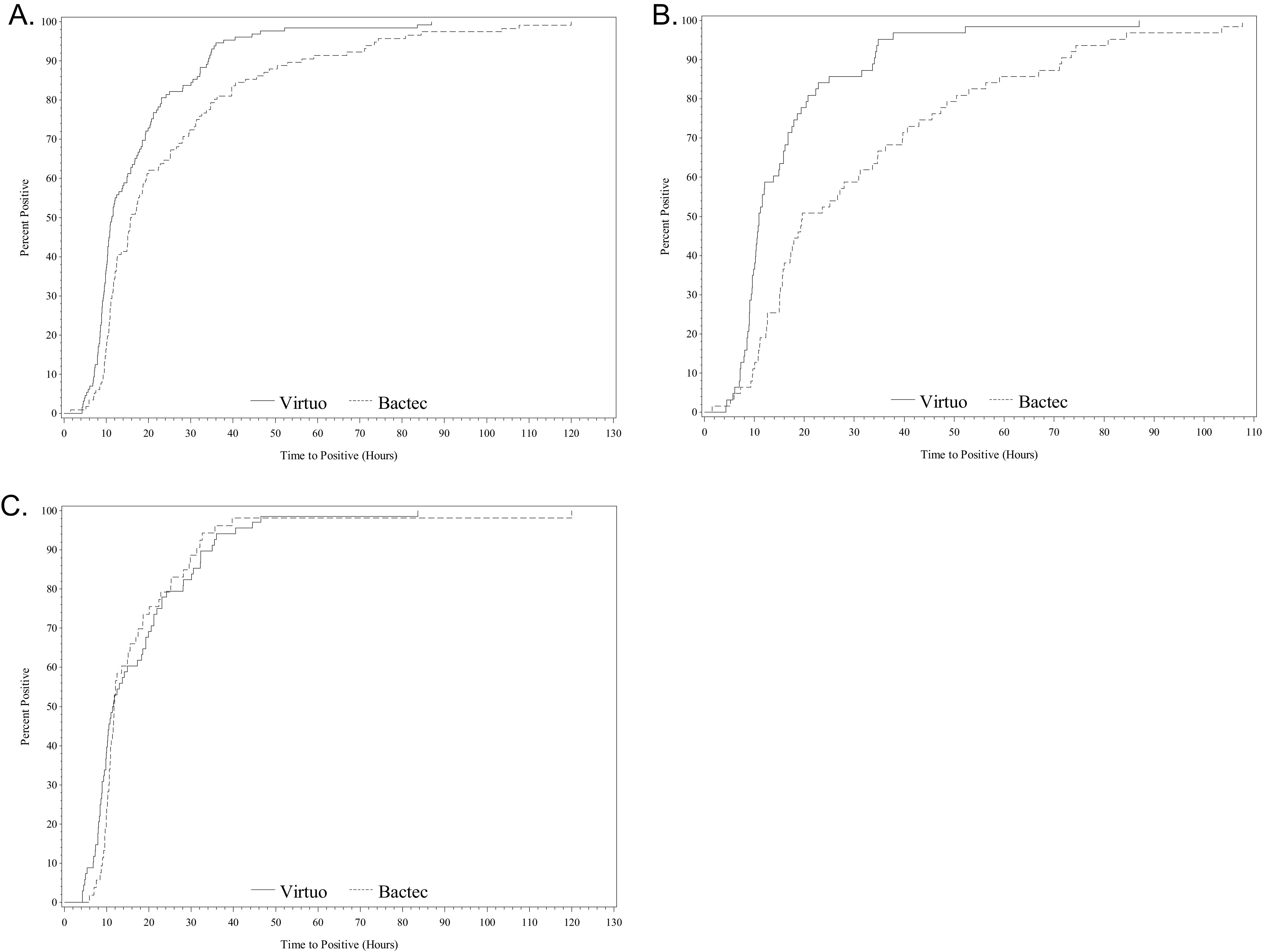
TTD by instrument, for significant isolates only for patients with previous antimicrobial treatment. (A) All bottles (Virtuo, *n* = 129; Bactec FX, *n* = 116) (*P* < 0.000). (B) Aerobic bottles (Virtuo, *n* = 63; Bactec FX, *n* = 63) (*P* < 0.0001). (C) Anaerobic bottles (Virtuo, *n* = 68; Bactec FX, *n* = 53) (*P* = 0.6749).

When considering patients with previous antimicrobial treatment, the TTD was significantly lower in the Virtuo arm for all bottle types. The median TTD was 8.7 h lower for aerobic bottles using Virtuo, while for anaerobic bottles, no significant differences were observed (Table 3 and Fig. 3B and C). For specific antibiotics, there was a shorter TTD for carbapenems and piperacillin-tazobactam with Virtuo. The first-line therapy antibiotics combined (amoxicillin-clavulanate, piperacillin-tazobactam, and carbapenems) showed a median TTD that was 11.2 (22.8 − 11.6) h shorter for samples incubated in the Virtuo system (*P* = 0.0003). Furthermore, the median TTD in this group was 15.6 (27.2 − 11.6) h shorter when considering aerobic bottles only (*P* < 0.0001).

**TABLE 3:** Time to detection by previous antibiotic treatment

Previously administered antimicrobial(s)	Type of bottle	No. of extractions; median TTD (h) (IQR)		*P* value^*a*^
		Virtuo	Bactec FX	
Any antimicrobial	All	129; 11.4 (9.0–20.8)	116; 15.9 (10.9–31.7)	**0.0001**
	Aerobic	63; 10.9 (9.1–18.6)	63; 19.6 (12.6–45.6)	**<0.0001**
	Anaerobic	68; 11.5 (8.6–22.5)	53; 11.7 (10.2–20.1)	0.6749

No antimicrobial	All	369; 10.2 (8.0–15.8)	364; 10.9 (8.9–15.1)	0.2254
	Aerobic	177; 11.5 (8.54–16.0)	121; 11.9 (10.0–18.2)	0.0572
	Anaerobic	192; 9.7 (7.7–15.4)	245; 10.3 (8.8–13.6)	0.4381

Carbapenems	All	24; 18.4 (11.6–21.7)	28; 31.2 (16.8–54.6)	**0.0016**
	Aerobic	17; 16.8 (10.9–22.3)	20; 34.2 (16.8–69.2)	**0.0052**
	Anaerobic	7; 19.9 (13.7–21.2)	8; 29.7 (18.8–34.1)	**0.0485**

Cephalosporins	All	14; 11.3 (10.1–32.3)	23; 13.6 (9.7–19.4)	0.8978
	Aerobic	9; 10.5 (9.9–15.9)	10; 15.0 (9.7–34.7)	0.2175
	Anaerobic	6; 21.4 (10.4–44.5)	13; 12.1 (10.6–16.9)	0.3180

Piperacillin-tazobactam	All	25; 9.5 (8.8–11.4)	26; 19.4 (12.6–39.7)	**<0.0001**
	Aerobic	13; 10.8 (9.1–12.0)	22; 18.0 (12.6–42.9)	**0.0043**
	Anaerobic	12; 9.1 (8.4–10.9)	4; 21.5 (17.6–25.3)	NA

Amoxicillin-clavulanic acid	All	15; 10.2 (8.0–36.0)	17; 11.6 (10.8–18.7)	0.7764
	Aerobic	4; 9.5 (5.9–49.3)	5; 30.9 (17.2–40.6)	NA
	Anaerobic	12; 8.7 (11.5–35.5)	12; 11.0 (10.5–14.6)	0.6411

First-line therapy (carbapenems, piperacillin-tazobactam, or amoxicillin-clavulanic acid)	All	64; 11.6 (8.9–20.5)	69; 22.8 (11.6–39.7)	**0.0003**
	Aerobic	34; 11.6 (9.1–20.4)	45; 27.2 (15.6–48.6)	**<0.0001**
	Anaerobic	31; 11.4 (8.5–20.6)	24; 16.3 (10.9–27.4)	0.1461

a Statistically significant values are indicated in boldface type. NA, not applicable due to the limited sample size.

### Workflow efficiency and hands-on time.

**FIG 4:**
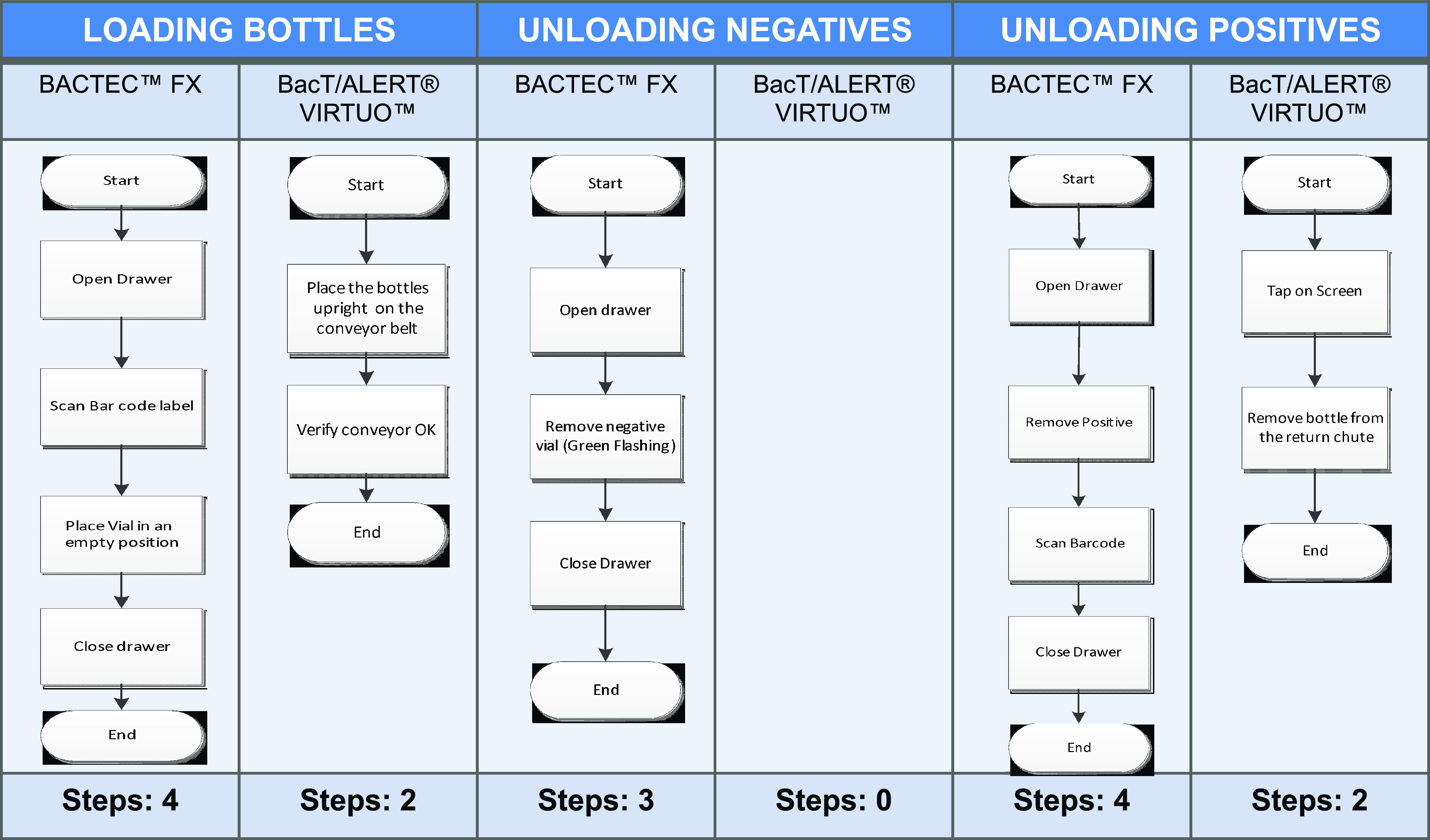
Number of steps required to process samples in each automated blood culture incubator. The steps are grouped for the following processes: loading of all bottles, unloading of negative samples, and unloading of positive samples. Unloading of negative bottles from the Virtuo system is automated; therefore, there are zero steps.

The mean times for loading each bottle by technicians were 7.28 and 1.32 s per bottle for Bactec FX and Virtuo, respectively. The mean times for unloading negative bottles were 1.89 s/bottle for Bactec FX and 0 for Virtuo, as the latter system automatically discards negative bottles. Nevertheless, for unloading positive bottles, Bactec FX demanded a mean time of 5.72 s/bottle, while Virtuo required 9.86 s/bottle. We had a median of 110 bottles loaded per day, with a positivity rate of 17%. According to our demand and positivity rate, also considering the time employed for maintenance, the hands-on time was shortened by 15 min per day when using Virtuo compared to Bactec FX (Table 4). Moreover, the number of manual steps when handling the bottles was reduced by 7 when using Virtuo compared to Bactec FX, with fewer manual steps to load bottles, unload negative bottles, and unload positive bottles (Fig. 4).

**TABLE 4:** Hands-on-time measurements

Parameter	Bactec FX		Virtuo		Mean time difference/bottle (min)
	Mean standard time (s)/bottle ± SD	Calculated total daily time (min)	Mean standard time (s)/bottle ± SD	Calculated total daily time (min)	
Bottles loaded/day (*n* = 110)	7.26 ± 3.7	13.31	1.32 ± 0.5	2.42	0.10
Positive unloaded bottles/day (17%)	5.72 ± 2.22	1.78	9.86 ± 2.88	3.07	−0.07
Negative unloaded bottles/day (83%)	1.89 ± 1.32	2.88	0	0	0.03
Daily maintenance	181	3.02	NA^*a*^	NA	3.02
Waste bin change every 72 bottles	NA	NA	8.48 ± 2	0.14	−0.14
Wkly cleaning of conveyor	NA	NA	12.25 ± 2.63	0.20	−0.20
Total		21.02		5.84	

a NA, not applicable.

## DISCUSSION

The time to inform a blood culture result has been reduced in recent years by the implementation of mass spectrometry (matrix-assisted laser desorption ionization–time of flight [MALDI-TOF]) and molecular approaches for species identification and the introduction of rapid AST directly from positive blood cultures. However, these novel rapid methods should also be accompanied by logistic improvements in the pre- and postanalytics so that the total time from the patient entering the hospital to reporting is reduced (11). In this clinical diagnostic trial, we focused on possible differences that could impact the time to detection before the microbiological analytical steps are done, including BC incubation.

Previous studies have shown a higher positivity rate or shorter TTD for Virtuo than for other automated incubation systems, but those studies were conducted either with a “before-and-after” design (12, 13), with spiked bottles (14, 15), or by duplicating samples in either aerobic or anaerobic bottle types (16, 17).

To our knowledge, we report the first clinical diagnostic study of this scale in a “real-world” setting with a crossover design comparing two automatic blood culture incubators using samples from patients with a suspected diagnosis of bacteremia/sepsis, as opposed to spiked vials. Our study design mimics that of clinical trials performed for drug marketing authorization, but patient randomization was replaced with the crossover design. With this approach, the patient characteristics in both arms were similar, avoiding potential biases that might affect the results and the need for duplicate samples.

The proportion of positive results was significantly higher for the samples incubated in the Virtuo system (18.2% versus 15.5% [*P* = 0.0003]), with similar numbers of false-positive results for both arms, although there were no significant differences when excluding the contaminating isolates (*P* = 0.0519). In a previous report, there was no statistical difference in recovery rates based on the overall concentration of microorganisms between the two BC systems (18).

Our primary outcome, TTD, was significantly shorter for the samples incubated in the Virtuo system for all bottles, with a median difference of 1.4 h. These results are in concordance with those of a previous study where the TTD for spiked samples was significantly shorter for aerobic and anaerobic bottles incubated in the Virtuo system (medians of 11.6 h and 10.1 h, respectively) than with Bactec FX (medians of 13.5 h and 12.2 h, respectively) (19). The TTD was also reported to be shorter for seeded bottles incubated in the Virtuo system, with 72.7% of organisms having a significantly shorter TTD for Virtuo than the ones incubated in the Bactec FX system (20).

In our study, this difference was due mostly to the reduced TTD for the aerobic bottles (difference in the median TTD of 3.8 h). This is likely related to a number of causes, but analysis of patients who were treated with antimicrobials prior to blood culture suggests that better neutralization may be one of them. The TTD is significantly shorter for bottles from subjects with previous treatment with antimicrobials (11.4 h for Virtuo versus 15.9 h for Bactec FX [*P* ≤ 0.0001]) and is shorter but not statistically significant for samples from patients without previous antimicrobial treatment (10.2 h for Virtuo versus 10.9 h for Bactec FX [*P* = 0.2264]). Previous publications report that over 40% of inpatients are administered antibiotics before blood collection for culture (21–23). In the present study, 25% of the samples positive for a significant organism were extracted after antimicrobial treatment was initiated. For these samples, the TTD was shorter for aerobic bottles, with a difference in the median TTD of 8.7 h (10.9 h for Virtuo versus 19.6 h for Bactec FX [*P* < 0.0001]), in favor of Virtuo. According to a previous study with spiked blood culture bottles (24), this difference for aerobic bottles could be explained by the better neutralization of antibiotics.

Regarding the workflow efficiency, the hands-on time was reduced by 15 min/day when using Virtuo, and the number of steps required to handle the bottles was reduced from 11 to 4, showing an optimization of preanalytical resources that can impact the results. These calculations are based on the number of bottles loaded per day and the proportions of positive/negative results in our laboratory. The time difference would be greater with a larger number of bottles loaded per day.

Our study has several limitations. First of all, molecular analysis was not performed for the false-positive samples to determine any uncultivable or fastidious organisms. However, the percentages of false-positive results were low and similar in both arms (20% for Virtuo and 17% for Bactec FX). Second, for logistic reasons, it was not possible to include all hospital wards from our institution, but with this sampling, we were able to include 90% of all blood cultures in the hospital. Third, the trial was performed in a single center.

The introduction of any improvement in the time to the detection of a positive blood culture should always be accompanied by effective logistics in the pre- and postanalytic steps. The “microbiologistics” (11), a term introduced by Lamy et al., are meant to include all of these aspects of the logistics in microbiology: the time to start the incubation of BC vials, the transport time, rapid communication of results, infectious disease consultation, or, even better, an antibiotic stewardship program. We have demonstrated different options to shorten the TTD, TAT, time to ID, and time to AST results, with changes in the automatic incubator, but these differences may not have all of their potential impacts if all of the other aspects of the diagnosis of bloodstream infection are not optimized. In the current study, we reported an overall median TAT ranging from 26.2 h to 28.3 h, suggesting that the time between positivity and reporting of Gram stain results is >10 h. In the analysis plan, the TAT was examined at the extraction level, including contaminants and multiple positive extractions during the same bacteremia episode. For these samples, the report is not immediate and might be delayed before validation. This may have led to an overall overestimation of the TAT. The enhancement of the workflow efficiency and the reduction in the hands-on time accomplished by using Virtuo could be part of the microbiologistics improvements.

In summary, we have shown in a real-world setting that Virtuo provides shorter TTD and TAT than Bactec FX and that the TTD for aerobic bottles is even shorter for patients who have received antimicrobials before blood culture extraction. This could have an important effect on the faster identification of causative microorganisms of BSIs and antimicrobial stewardship.

## MATERIALS AND METHODS

### Study design.

We compared the performance of BacT/Alert Virtuo (bioMérieux, France) to that of the Bactec FX instrument (BD, USA) with a prospective crossover diagnostic clinical trial.

We included 3,625 nonpediatric patients (≥16 years old) suspected of having bacteremia/fungemia who were localized in different wards of Ramón y Cajal Hospital (Madrid, Spain): the emergency department (ED), internal medicine, hematology, surgical intensive care unit (ICU), medical ICU, general surgery, and infectious diseases. Since 70% of all blood cultures of the hospital are routinely ordered from the ED, we divided the patients into two strata: stratum 1 was assigned to the ED, and stratum 2 included all other wards. Initially, each stratum was randomly assigned to one of the incubators and then alternated every 2 weeks for 6 months (from November 2018 to April 2019).

BC testing was performed using Bactec Plus Aerobic/F bottles (aerobic bottles) and Bactec Lytic/10 Anaerobic/F bottles (anaerobic bottles) incubated in the Bactec FX system or using BacT/Alert FA Plus bottles (aerobic bottles) and FN Plus bottles (anaerobic bottles) incubated in the Virtuo system. Each sample, obtained from one blood extraction (one blood draw, from either venipuncture or catheter), was incubated in both aerobic and anaerobic bottles, except when indicated otherwise (Table 5). Sample and extraction are used as synonyms. Two (or, in select patients, three) different samples that were obtained at the same time were defined as a blood culture set. A set therefore typically included 4 bottles, 2 aerobic and 2 anaerobic, from two different blood extractions. When 3 extractions were required, a set included 6 bottles. All bottles were processed according to the laboratory’s routine standard operating procedure from the moment that they were flagged positive. Samples were incubated in either system for a maximum of 5 days according to the standard of care. No additional samples were required from the patients.

**TABLE 5:** Summary of samples and types of bottles analyzed

Parameter^*a*^	Value for system		*P* value^*c*^
	Virtuo	Bactec FX	
No. of blood culture sets	2,391	2,588	
No. of extractions evaluated (%)	4,797	5,160	
All bottles negative	3,923 (81.8)	4,361 (84.5)	
At least 1 bottle positive (including contaminants)	874 (18.2)	799 (15.5)	
No. of aerobic bottles analyzed/set (%)			
0	3 (0.1)	7 (0.3)	
1	90 (3.8)	125 (4.8)	
2	2,219 (92.8)	2,390 (92.4)	
3	79 (3.3)	66 (2.6)	
No. of anaerobic bottles analyzed/set (%)			
0	5 (0.2)	8 (0.3)	
1	78 (3.3)	124 (4.8)	
2	2,229 (93.2)	2,391 (92.4)	
3	79 (3.3)	65 (2.5)	
No. of 1st bottles detected as positive (%)			0.2657
Aerobic	466 (53.4)	391 (48.9)	
Anaerobic	402 (46.1)	405 (50.7)	
Both simultaneously	3 (0.3)	3 (0.4)	
Unknown	2 (0.2)	0	
No. of positive samples/total no. of samples (%)	874/4,797 (18.2)^*b*^	799/5,160 (15.5)	**0.0003**
No. of positive samples/total no. of samples (%) excluding false positives	854/4,797 (17.8)	782/5,160 (15.2)	**0.0003**
No. of false-positive samples	20	17	
No. of samples with contaminant	354	302	
No. of samples with significant organism	502	480	
No. of positive aerobic samples/total no. of aerobic samples (%)	470/4,765 (9.9)	394/5,103 (7.7)	**0.0002**
No. of positive aerobic samples/total no. of aerobic samples (%) excluding false positives	459/4,765 (9.6)	381/5,103 (7.5)	**0.0001**
No. of false-positive samples	13	13	
No. of samples with contaminant	217	197	
No. of samples with significant organism	240	184	
No. of positive anaerobic samples/total no. of anaerobic samples (%)	406/4,773 (8.5)	408/5,101 (8.0)	0.3593
No. of positive anaerobic samples/total no. of anaerobic samples (%) excluding false positives	399/4,773 (8.4)	404/5,101 (7.9)	0.4246
No. of false-positive samples	7	4	
No. of samples with contaminant	137	106	
No. of samples with significant organism	262	298	

a Seven cultures had aerobic and anaerobic bottles positive at the same time. They are included in both the aerobic and anaerobic portions of the table.

b One culture in the Virtuo system was missing bottle type data for two extractions.

c Statistically significant values are indicated in boldface type.

The study was approved by Hospital Universitario Ramón y Cajal ethics committee (reference number 332-17), and patient informed consent was waived.

### Blood culture processing.

All processes were performed by trained technicians using both systems, according to the same workflow after a BC bottle was flagged as positive: (i) Gram stain and subculture on blood agar and chocolate agar, (ii) identification directly from the pellet by MALDI-TOF mass spectrometry (Bruker, Germany) (25), (iii) rapid disc diffusion antibiogram, and (iv) final AST by a semiautomated microdilution system (MicroScan WalkAway; Beckman Coulter, USA). Gram stain, MALDI-TOF mass spectrometry, and AST results were communicated to the attending physician immediately if the microorganism was not considered to be a contaminant and if this was the first bacteremia episode for that patient.

### Time measurement definitions.

**FIG 5:**
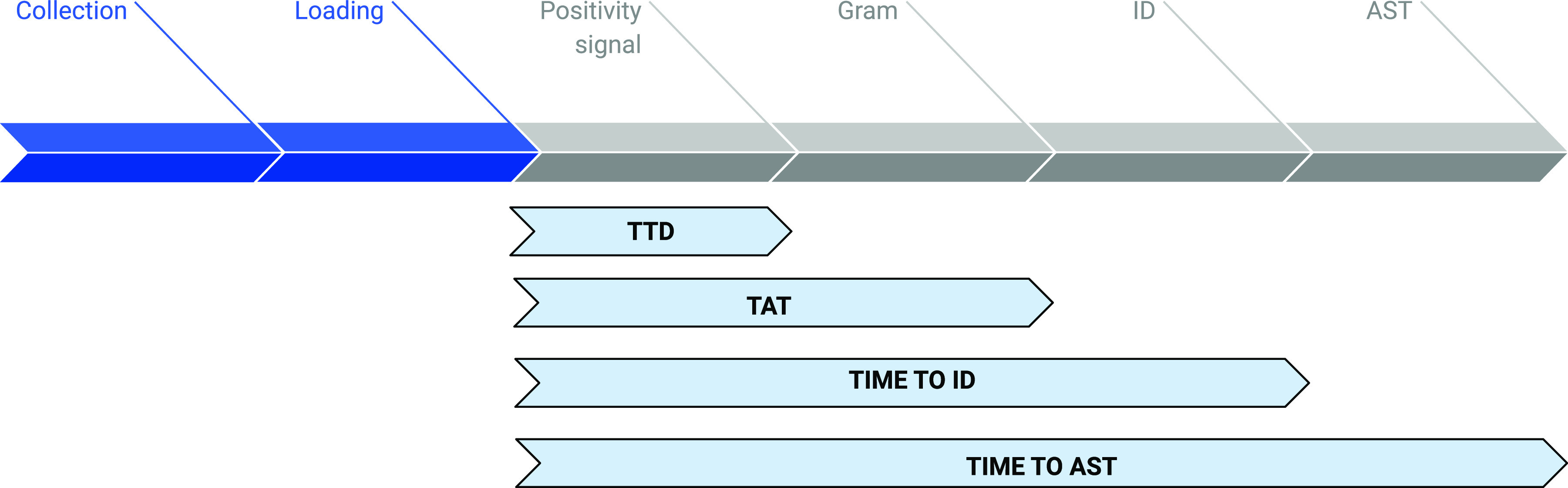
Schematic representation of different time measurements. TTD, time to detection; TAT, turnaround time; ID, identification; AST, antimicrobial susceptibility testing.

The following definitions were used (Fig. 5): (i) time to detection (TTD), the time from the loading of the sample into the automated blood culture incubator to a positivity signal for the first bottle, either aerobic or anaerobic (if the anaerobic and aerobic bottles were positive at the same time, both were included); (ii) turnaround time (TAT), the interval from loading of the specimen into the automated blood culture incubator to the reporting of the Gram stain results to the clinician; (iii) time to identification (time to ID), the time from the loading of the sample into the automated blood culture incubator to identification; and (iv) the time to AST results, the time from the loading of the sample into the automated blood culture incubator to the final AST report.

### Data analysis.

The time for loading into the incubators, TTD, TAT, time to ID, and time to AST results were retrieved from the laboratory’s information system and patient clinical charts and recorded in an electronic database.

As a general rule, the following microorganisms, when present in a single sample with no other cultures positive for the same organism within 96 h, were considered contaminants: coagulase-negative staphylococci (CoNS) with the exception of Staphylococcus lugdunensis, *Propionibacterium* spp., *Bacillus* spp. different from Bacillus anthracis, *Corynebacterium* spp. (diphtheroids), *Aerococcus*-like organisms, *Micrococcus* spp., viridans group streptococci, *Neisseria* spp. excluding Neisseria gonorrhoeae or N. meningitidis, and *Streptomyces* spp. Furthermore, all positive results were reviewed by clinical microbiologists and medical doctors along with the patient’s clinical history to differentiate contaminants from significant microorganisms.

Antibiotic treatment was recorded for all patients considered to have a significant isolate producing bacteremia/fungemia. Previous antibiotic treatment was defined as any antimicrobial administered in the 5 days preceding sample extraction.

False-positive results were defined as bottles that signaled “positive” by the instruments but with no growth after subculture.

### Workflow efficiency and hands-on-time measurement.

The standard time (hands-on time) and the number of steps required when processing either a negative or a positive BC with the respective automated systems were measured, including all of the manipulations performed by the technician, according to the instructions provided by the instrument user manuals.

Two technicians were observed from 8 to 9 a.m. and from 1 to 3 p.m. Monday to Friday for 4 weeks. Time was measured as follows:

- Loading time for one bottle
  - Bactec FX: starting from the technician being located in front of the machine and finishing when the drawer is closed.
  - Virtuo: starting from the technician being located in front of the machine and finishing when the bottle is placed on the conveyor.
- Unloading time for one positive bottle
  - Bactec FX: starting from the technician being located in front of the machine and finishing when the drawer is closed.
  - Virtuo: starting from the technician pressing the “remove positive bottle” icon and finishing when the bottle is removed.
- Unloading time for one negative bottle
  - Bactec FX: starting from the technician being located in front of the machine and finishing when the drawer is closed.
  - Virtuo: none.

Sampling was done to estimate the minimum number of observations to have a statistically relevant result for the hands-on-time comparison.

The technicians were observed for a period of 20 days until we reached the following number of observations:

- Loading BacT/Alert Virtuo bottles, 340 observations
- Unloading BacT/Alert Virtuo bottles (positive), 181 observations
- Unloading BacT/Alert Virtuo bottles (negative), automated process, 0 observations
- Loading Bactec FX bottles, 431 observations
- Unloading Bactec FX bottles (positive), 101 observations
- Unloading Bactec FX bottles (negative), 233 observations

The mean time employed and the standard deviation (SD) were calculated, and the total time employed per day was estimated for a mean activity of 110 bottles incubated in each instrument per day.

### Statistical analysis.

Categorical variables were compared using a chi-squared test. Continuous variables were compared using a *t* test. Time-to-event variables were compared using a Tarone-Ware test. The most common statistics used to measure the differences between time-to-event curves are the log rank and Wilcoxon tests. The log rank test emphasizes primarily the differences between the curves at the longest times to event, while the Wilcoxon test emphasizes those at the shortest. The Tarone-Ware test used here looks for symmetry in the behaviors of the curves around the median.

All statistical analyses were performed using SAS version 9.4 (SAS Institute, Cary, NC, USA). *P* values of ≤0.05 were taken to indicate significant differences.
